# Pneumothorax in lung cancer following anlotinib treatment: A case report

**DOI:** 10.1097/MD.0000000000029273

**Published:** 2022-07-22

**Authors:** Lei Yang

**Affiliations:** Department of Respiratory and Critical Care Medicine, The First People's Hospital of Neijiang, China.

**Keywords:** adverse drug reaction, anlotinib, lung adenocarcinoma, pneumothorax

## Abstract

Anlotinib is an oral tyrosine kinase inhibitor targeting vascular endothelial growth factor receptor, and has been approved for the treatment of patients with advanced nonsmall cell lung cancer who had received at least 2 previous chemotherapy and epidermal growth factor receptor/anaplastic lymphoma kinase targeted therapy regimens. Pneumothorax is a rare adverse reaction of this drug. Here, we present a case of pneumothorax following anlotinib treatment in a patient with lung cancer.

**Patient concerns:**

A 61-year-old Asian male long-term smoker was admitted to the hospital in November 2019 with sputum production and dyspnea.

**Diagnosis:**

The patient was diagnosed with right lung adenocarcinoma with mediastinal and rib metastases, combined with chronic obstructive pulmonary disease and pulmonary bullous disease.

**Interventions:**

The patient was treated with oral administration of anlotinib. The patient had a recurrent pneumothorax that improved after drug withdrawal and was free of recurrence. Therefore, pneumothorax caused by rupture of the pulmonary bullous due to anlotinib was considered.

**Outcomes:**

After discontinuing anlotinib, the patient has not developed pneumothorax to date.

**Lessons:**

Pneumothorax may occur when VEGF is inhibited, which can promote the proliferation and repair of alveolar wall substances, leading to alveolar rupture. With respect to pneumothorax, it is necessary to be aware of the risk of pulmonary bullous rupture during antitumor treatment with small-molecule tyrosine kinase drugs.

## 1. Introduction

According to global cancer statistics for 2020,^[1]^ although breast cancer has surpassed lung cancer to become the world's largest number of tumors, lung cancer still has the highest mortality rate, with the number of new cases reaching 2.2 million, leading to approximately 1.8 million cases. More than 80% of lung cancers are nonsmall cell lung cancers (NSCLC). Anlotinib is a small-molecule tyrosine kinase inhibitor that inhibits vascular endothelial growth factor receptor 2/3, fibroblast growth factor receptor 1–4, platelet-derived growth factor receptor B α/β. Based on the clinical trial ALTER-0303,^[2]^ (National Medical Products Administration) approved anlotinib as a third-line treatment for patients with advanced NSCLC in China. Anlotinib (12 mg/d) was administered orally for 14 consecutive days, followed by 7 days of withdrawal and a 21-day cycle. Spontaneous pneumothorax is uncommon in anlotinib-targeted therapy, but severe adverse reactions often lead to discontinuation and hospitalization of patients.

## 2. Case presentation

**Figure 1.**
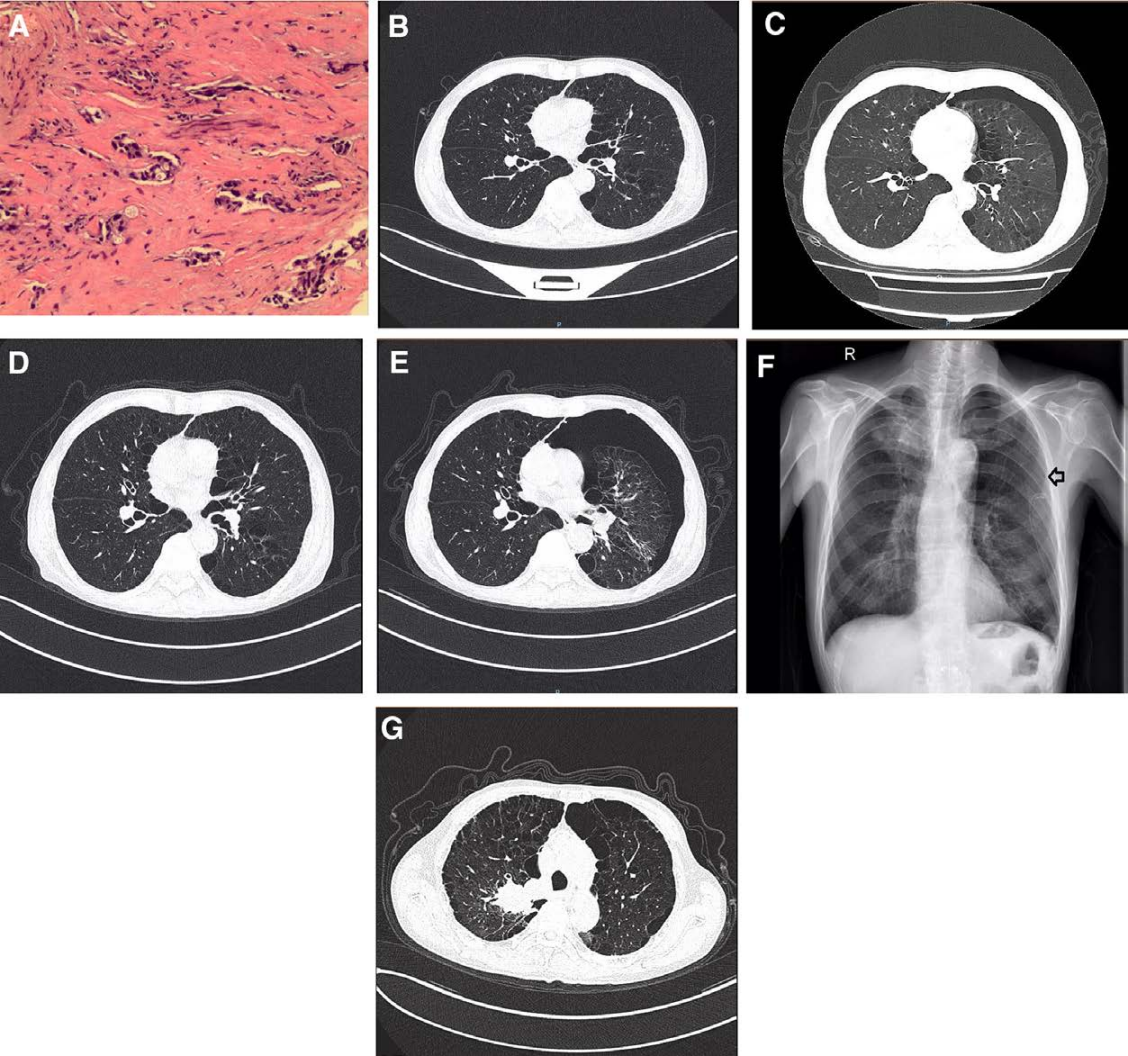
(A) Pathological diagnosis of lung adenocarcinoma; (B) contrast chest CT before anlotinib treatment; (C) contrast chest CT showing the first pneumothorax; (D) contrast chest CT showing pneumothorax absorption after best supportive care; (E) contrast chest CT showing second pneumothorax. (F) Chest X-ray: pneumothorax absorption after closed thoracic drainage; (G) contrast chest CT: no pneumothorax occurred during follow-up. CT = computed tomography.

A 61-year-old Asian male long-term smoker was admitted to our hospital in November 2019 with sputum production and dyspnea. The patient was diagnosed with right lung adenocarcinoma (Fig. 1A) with mediastinal metastasis and rib metastasis at T3N2M1 stage IVA, positive for tp53 and negative for epidermal growth factor receptor/anaplastic lymphoma kinase/ROS proto-oncogene 1, receptor tyrosine kinase/Kirsten rat sarcoma viral oncogene combined with chronic obstructive pulmonary disease (COPD), pulmonary bullous. Contrast computed tomography (CT) scan of the chest revealed a 5.8 × 5.1 × 6.6 cm right upper lobe mass with mediastinal lymphadenopathies. There were no abnormalities on physical examination except for chest examination, in which breathing sounds were diminished. The patient refused immunotherapy for economic reasons, and the lesions continued to progress after bevacizumab and multiline chemotherapy. The patient was re-examined on May 26, 2021 (Fig. 1B), which indicated that the lesion had enlarged. After communication, the patient was treated with oral administration of anlotinib (12 mg d1–d14, PO q21d) on July 07, 2021, and was followed up outside the hospital. The patient was admitted to the hospital on October 26, 2021 with dyspnea (grade 3),^[3]^ oral ulcer (grade 2), and anorexia (grade 2). Chest CT revealed left pneumothorax (Fig. 1C). Physical examination revealed that the breathing sounds on the left chest wall significantly disappeared, and the percussion sound was tympanic. Considering the symptoms of pneumothorax, anorexia, and oral ulcers, anlotinib was discontinued after the communication. After the best supportive treatment, chest CT showed pneumothorax absorption (Fig. 1D) on November 04, 2021. Due to the stability of pulmonary lesions, patient was required to continue oral administration of anlotinib (anlotinib 12 mg d1–d14, PO, q21d) on November 07, 2021. The patient experienced dyspnea again on November 13, 2021, and chest CT (Fig. 1E) confirmed left pneumothorax. Anlotinib was discontinued and the most supportive treatment was ineffective. The symptoms improved after closed thoracic drainage on November 17, 2021 (Fig. 1F). The patient was discharged home to rest for 1 month before continuing treatment. He is currently undergoing immunotherapy. The patient had a history of smoking and pulmonary bullous disease. The patient has had no history pneumothorax since the diagnosis of lung cancer and prior to anlotinib intake. After discontinuing anlotinib, the patient has not developed pneumothorax to date (Fig. 1G).

## 3. Discussion

Anlotinib is an oral small-molecule tyrosine kinase inhibitor that was independently developed in China. It was approved for third-line treatment of advanced NSCLC in May 2018. Common adverse reactions to anlotinib include elevated blood pressure, fatigue, hand–foot skin reactions, and diarrhea. Pneumothorax is a rare adverse reaction to this drug.^[4]^ Although the patient had a history of pulmonary bullous, there was no history of pneumothorax attack since 2019 when he was diagnosed with pulmonary adenocarcinoma and before taking anlotinib. Pneumothorax appeared after the administration of anlotinib, and the symptoms improved after the discontinuation of anlotinib. Anlotinib rechallenge again resulted in pneumothorax. These findings suggest that spontaneous pneumothorax was associated with anlotinib. In previous clinical trials, ALTER-0303^[2]^ indicated that pneumothorax a rare complication of anlotinib, and 2 patients discontinued treatment due to pneumothorax. This study did not further analyze the mechanism of pneumothorax or the clinical characteristics of patients. Pneumothorax may be related to the pleural invasion of pulmonary metastasis tumor necrosis, and cavity formation caused by targeted therapy. Other analyses,^[5–7]^ reported that use of multi-target tyrosine kinase inhibitors, is associated with spontaneous pneumothorax and cavitary and pleural-based lung lesions in metastatic sarcoma. This may provide some insight into the mechanism underlying the development of pneumothorax, which has yet to be clearly defined in this population. It has been suggested that cytotoxic agents may induce necrosis and cavitation of lung nodules, thereby increasing the risk of rupture and development of pneumothorax. In addition to anlotinib, other chemotherapeutic agents have been reported to be associated with pneumothorax in patients with advanced sarcomas. This theory may be the cause of the lung metastasis pneumothorax, but cannot be considered the cause in this patient.

Two-thirds of the patients with lung tumor had a history of smoking,^[1]^ and COPD is a common comorbid disease or lung bullous.^[8]^ It is necessary to consider that pneumothorax can occur in bullous and COPD patient. However, there is also a risk of anlotinib-induced pulmonary bullous rupture. But this cannot be explained-by pleural invasion of pulmonary metastasis, tumor necrosis, or cavity formation caused by targeted therapy. Pneumothorax may occur when VEGF is inhibited. Alveolar rupture may occur, resulting in pneumothorax, which is similar to gastrointestinal perforation.^[9]^ Based on the experience with our patient, it is necessary to be aware of the risk of pulmonary bullous rupture during antitumor treatment with small-molecule tyrosine kinase drugs. At present, anlotinib combined with chemotherapy, radiotherapy, and immunotherapy has been tested in several clinical trials as a multi-tumor first-line treatment. Therefore, attention should be paid to prevention of pneumothorax in the clinical practice.

## Acknowledgments

We would like to thank the anonymous reviewers for their helpful remarks.

## Author contributions

Data curation: Yang Lei

Writing – original draft: Yang Lei

Writing – review & editing: Yang Lei
